# A Tailored Ontology Supporting Sensor Implementation for the Maintenance of Industrial Machines

**DOI:** 10.3390/s17092063

**Published:** 2017-09-08

**Authors:** Elaheh Maleki, Farouk Belkadi, Mathieu Ritou, Alain Bernard

**Affiliations:** 1 LS2N (UMR CNRS 6004): Ecole Centrale de Nantes, 44300 Nantes, France; Farouk.Belkadi@ls2n.fr (F.B.); Alain.Bernard@ls2n.fr (A.B.); 2 LS2N (UMR CNRS 6004): University of Nantes, 44035 Nantes, France; Mathieu.Ritou@univ-nantes.fr

**Keywords:** industrial machinery maintenance, ontology-based model, sensors implementation

## Abstract

The longtime productivity of an industrial machine is improved by condition-based maintenance strategies. To do this, the integration of sensors and other cyber-physical devices is necessary in order to capture and analyze a machine’s condition through its lifespan. Thus, choosing the best sensor is a critical step to ensure the efficiency of the maintenance process. Indeed, considering the variety of sensors, and their features and performance, a formal classification of a sensor’s domain knowledge is crucial. This classification facilitates the search for and reuse of solutions during the design of a new maintenance service. Following a Knowledge Management methodology, the paper proposes and develops a new sensor ontology that structures the domain knowledge, covering both theoretical and experimental sensor attributes. An industrial case study is conducted to validate the proposed ontology and to demonstrate its utility as a guideline to ease the search of suitable sensors. Based on the ontology, the final solution will be implemented in a shared repository connected to legacy CAD (computer-aided design) systems. The selection of the best sensor is, firstly, obtained by the matching of application requirements and sensor specifications (that are proposed by this sensor repository). Then, it is refined from the experimentation results. The achieved solution is recorded in the sensor repository for future reuse. As a result, the time and cost of the design process of new condition-based maintenance services is reduced.

## 1. Introduction

The quality of a product is highly affected by its process parameters [1]. Several methods are presented in the literature, mostly connecting product quality to an analysis of the production process. This can be determined by two different approaches: a posteriori direct inspection (such as three-dimensional (3D) measurement of a machine or its surface topology [2]); or indirect assessment by in-process monitoring. Many of these methods use the data acquired from the product quality analysis to define the functional characteristics and the health status of machines involved in the production process. So, to maintain the optimal performance that guarantees best product quality, industrial machinery passes throughout a transition from the traditional ‘fail-and-fix’ to ‘predict-and-prevent’ e-maintenance-based strategies [3]. More specifically, maintenance strategy has evolved from corrective maintenance to the Condition-Based Maintenance (CBM), which is based on real-time monitoring of the machine and the produced products [4].

As a result, adopting new tools and methods to meet the challenges in front of this new strategy has received considerable attention from both academic and industrial actors. The new approaches propose to adopt these tools and methods through lifecycle collaborative development supported by the integration of a product, a service, a sensor, and the internet [5]. As a result, capturing information from continuous health monitoring and analyzing data from products and services during their lifecycle is crucial [6]. Thus, the new form of industrial maintenance is highly dependent on the data collection capabilities of the both product and machine. In this challenging context, maintenance in the industrial machinery should take benefits from the burgeoning advancements in the field of Cyber-Physical Systems (CPS) for integrating ICT (Information and Communications Technology) in life cycle engineering [7].

Additionally, industrial machinery has evolved to mechatronic systems [8]. In this context, while the role of the sensor in the mechatronic system is “to make the system respond correctly in different conditions” [9], its role in preventive maintenance is to support a “guaranteed solution for trouble-free operations” [10]. As a result, the aim of original equipment manufacturers (OEMs) is not only to increase the technological performance of their products. It is also to improve the capability of information capture to guarantee the solution for the customer. This object will be fulfilled by continuous monitoring of a machine’s health. In this point of view, adding CPS to the product is not a part of product development but part of a service support system.

Considering all of the above, sensors are considered to be at the core of new industrial machinery maintenance due to their ability to enable lifecycle observation. Besides, monitoring a machine during its lifecycle (from design to the usage and end of life) makes it crucial to adopt an interdisciplinary design approach [11]. In practice, several sensors are proposed from various providers for the same measure, and in many cases with the same nominal specification. However, the real performance varies from one sensor to another (and sometimes for the same sensor) depending on the working conditions and desired service requirements. Finding the best couple of sensors to capture the desired measures with the minimum cost and the maximum efficiency is complicated. More challenges arise during the integration of various modules of a machine, a service, and sensors, which requires collaborative action between various experts.

Thus, it is crucial to provide engineers with robust tools which simplify a sensor’s selection and integration process. There are many studies regarding the applications of sensor networks and the use of ontology in an industrial context [12]. However, these few samples show that these ontologies are developed within a specific application with a limited scope. Also, sensor ontologies are generally developed according to the data provided by specific sensor providers. Consequently, the generalization of attributes is not easy. Tailoring sensor ontology for the design of a maintenance service requires considering not only the theoretical attributes as specified by the sensor provider but also the real characteristics as identified during the usage phase. This approach facilitates the linkage between sensor state and risk of breakdown symptoms.

In this context, the interest of this study is to highlight the role of sensors in Condition-Based Maintenance (CBM). The second interest of this study is to propose a tailored ontology for sensors to support the search for, sharing, and reuse of knowledge during the design of a maintenance service. To provide a new CBM, a primary list of suitable sensors, according to the use case constraints, could be obtained from an ontology-based repository. This repository describes the nominal characteristics of sensors. Engineers can refine this selection based on the real performances of the sensors resulting from previous experiments stored in the same repository.

The scope of this paper is the description and validation of the proposed sensor ontology. To do so, the remainder of this paper is organized as follows. Section 2 provides a literature review to describe the role of a sensor and its ontology in industrial services. Then, a Failure Mode, Effects, and Criticality Analysis (FMECA)-based method is proposed for the choice and implementation of sensor, for the design of a maintenance service. Section 3 describes the global model of the proposed ontology. It also details the design methodology and the structure of the proposed ontology. Section 4 presents the application of the sensor ontology in an industrial case using Protégé software in order to validate the completeness and the utility of the proposed ontology. Finally, Section 5 offers conclusions and possible exploitation of this ontology.

## 2. Need of Sensor Ontology

### 2.1. Role of Sensors in Industrial Services

Entering into the field of software-based smart services, tool makers evolved to service providers. While the focus of tool makers is on primary services, service providers upgrade the level of service to “online monitoring, condition based maintenance, and an availability guarantee” [13]. Lifecycle monitoring and providing customized services are some differences between traditional manufacturing systems and maintenance service providers [14].

Consequently, to cope with the competitive market of maintenance service in industrial machinery, OEMs need to bear in mind some key considerations about information capturing mechanisms [7]. To offer the above-mentioned integrated system of product, service and sensor, several actors from various disciplines are involved in the design process.

In this context, it is crucial to ensure semantic interoperability and efficient communication between the various stakeholders involved during both the system’s implementation and execution. This could be fulfilled by supporting the design process with domain knowledge as a semantic backbone adjusted to the maintenance design. The commonly accepted approach for structuring the domain knowledge is constructing domain ontologies to model concepts and relationships [15]. In other words, to facilitate data exchange between engineering activities in collaborative design, ontology-based approaches have been proposed in the literature [16].

An ontology is defined as “a set of concepts and relationships used to describe a particular domain of knowledge” [17]. Relatively, ontology-based modelling is a well-known approach to support knowledge integration and interoperability between information technology (IT) systems during collaborative business process [18]. The ONTOlogy for Product Data Management (ONTO-PDM) framework is an example of such solutions to allow information exchange and interoperability between enterprise applications [19]. Moreover, to cope with the complexity of engineering knowledge, modularity in ontology design is proposed as a promising approach [20]. Modular ontology development proposes that rather than having a massive ontology to cover a domain, it is necessary to abstract and generalize concepts into separate ontologies. This will be useful to allow better reusability, flexibility, and maintainability.

### 2.2. Sensor Implementation in the Maintenance Service Design Process

To enable a sensor-based maintenance strategy, engineers from multidisciplinary domains interact to select the optimal solution for maintenance service design and implementation. The maintenance is designed according to the characteristics of the target production system and its context of use as specified by the customer [21]. Thus, engineers need methods and tools to consistently manage the huge quantity of shared information during the design and implementation processes of the maintenance strategy.

Using knowledge-based systems to support the development of smart sensors has often been introduced in the literature. The main role of a knowledge base is to support the finding and reusing of relevant knowledge [22]. Knowledge-based frameworks have recently been implemented to manage the collaborative functioning of a set of sensors and the utilization of the measured data depending on the context of use during the exploitation stage [23].

**Figure 1:**
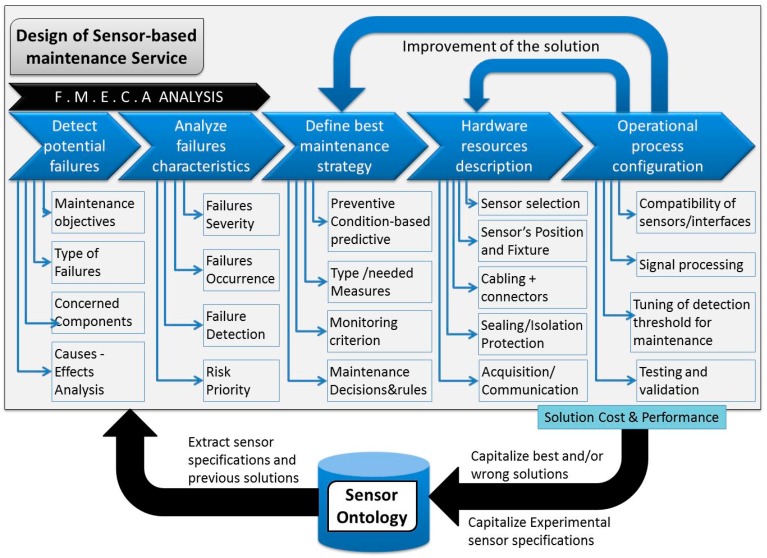
Sensor implementation process for maintenance and monitoring perspective. FMECA: Failure Mode, Effects, and Criticality Analysis.

Figure 1 shows the proposed empirical process for the identification and the implementation of the best sensors able to support a CBM strategy. FMECA (Failure Mode, Effects and Criticality Analysis) is the first step to be performed at the product or component level to detect the potential risk of failures as well as the concerned components along a machine’s lifecycle. Additional analyses are then made as a part of the FMECA process. The analyses identify the main causes–effects and the global risk priority number (RPN) for each detected failure based on the evaluation of the related failure severity, occurrence, and detection indicators [24]. Based on the machine’s characteristics and history and considering the failure type and RPN, experts have to adopt a suitable maintenance strategy for each critical failure. In this third step, a continuous monitoring process has to be defined to support either condition-based or predictive or preventive maintenance. This includes the identification of the needed measures, monitoring criterion, and decision rules to be applied to resolve machine health problems. Remaining Useful Life (RUL) is one of the most significant indicators generally used to evaluate the time length from the current instant to the end of the useful life of the system [25]. The implementation of the planned maintenance strategy consists in the identification of suitable types of sensors for the needed measures and to check the possibility of using already installed sensors in the machine. This requires verifying the possible measuring technology for the measurement of any specific information (Figure 1).

The detailed design stage concerns how to configure all hardware resources (e.g., sensors) as well as how to arrange and handle identified service information, etc. For instance, the following questions need to be answered when defining a potential integration solution:

- What sensor specification is needed to support measuring performance, depending on the product’s working conditions?
- What additional equipment is needed to support the functioning of the selected sensors?
- What are the main potential positions of sensors regarding the product’s structure for maximum measuring performance?
- What is the ideal fixture system for connecting sensors to the product’s components?
- What is the expected performance of the selected sensor following a specific product–service configuration?
- What type of data processing and analysis method is needed?

The integration solution could also include useful information about the potential assembly steps to connect all sensors and equipment to a machine’s components. The last stage is the configuration and validation of all operational conditions to have the maintenance solution ready for use. The first critical question is the verification of compatibility and interfacing between sensors as well as between sensors and machine components. Mechanical and electrical behaviors as well as signal processing and a sensitivity analysis are examples of the performed tests. The last configuration step is the tuning of detection thresholds necessary to trigger maintenance decisions during the machine’s usage life stage.

Following a knowledge-based approach, during the design process, the engineers have to connect to the knowledge repository in order to extract and reuse useful knowledge. Knowledge-based design is a process to reuse capitalized knowledge from past experience in new projects [26]. It is also conducted through knowledge sharing between involved stakeholders to ensure common representation of the problem and consistency of the final solution [27]. By using a sensor repository, engineers can obtain rapid access to the detailed specifications of a variety of sensors as provided by the related manufacturer. They also have access to the real specifications capitalized from past “in situ” experimentations. The measurement requirements will be matched with the sensors’ capabilities and the machine’s working conditions with the sensor’s recommended working conditions as specified in the repository. The result of this matching supports the selection of the best sensor for every service-needed measure. To be efficient, the sensor repository should be organized in a consistent way to facilitate searching and queries. The authors consider this as the main role of the proposed sensor ontology.

## 3. Proposition of a Sensor Ontology Structuring the Knowledge Repository

There is a wide range of information resources for sensor specification, from commercial to industrial and academic references, when considering that a general sensor’s definition and capabilities could make the basis for building an ontology. Sensors are able “to offer large potential to sense, store, and analyze equipment data to predict health status” [28]. This general foundation must be customized according to the special context of predictive maintenance.

However, despite the increasing technological efficiency, the lack of formal representation of sensor-measured data limits us taking advantage of the semantic quality of the information provided by new sensors. It also limits the capacity to exploit relevant knowledge for daily business activities. There are some sensor ontologies covering a specific domain, such as the ontology-based model for data acquired from recognition algorithms through light wave technology proposed by [29] dedicated to camera and vision sensors. Sensor description is also considered in the OntoPhil developed by [30] used as an intermediate support for communication between agents involved in a smart city’s environment.

In this paper, the ambition is to target a generic ontology to be used in the design and implementation of a sensor-based maintenance process (i.e., structuring the knowledge repository of Figure 1). Taking advantages from its generic property, this ontology could be also used to support the creation and the management of other kinds of sensor-based industrial processes, such as the continuous control of a production system. The next section describes the methodology and the main modules of the proposed ontology.

### 3.1. Methodology of Sensor Ontology Design

**Figure 2:**
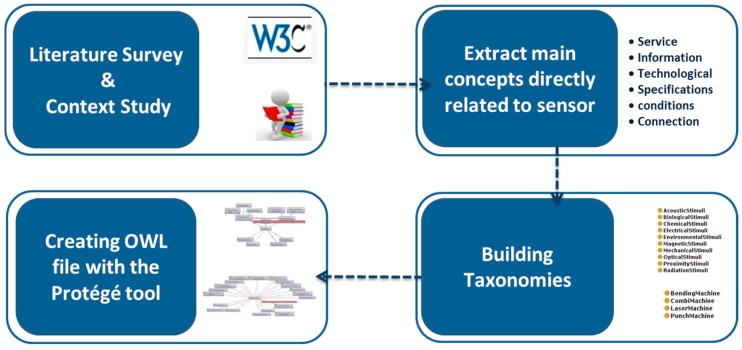
Methodology to build the sensor ontology. OWL: web ontology language.

The proposed ontology is developed with an operational perspective based on a literature survey and analysis of the project’s functions (Figure 2). The first step for ontology building is the analysis of engineering practices during the design process in terms of knowledge and information exchange. Then, the sensor ontology is created based on the literature survey and commercial information to capture the domain knowledge and currently developed ontologies about sensors [31]. Using related handbooks and the W3C Semantic Sensor Network Incubator Group (SSN-XG), we extracted the elements, features, and characteristics of sensors. These elements are extended and classified to connect sensor specification to different facets of maintenance service design and implementation. SSN uses the “Stimulus-Sensor-Observation ontology design pattern”, which is a “generic and reusable component for all kinds of observation-related ontologies” and “introduces the key classes and their relations such as stimuli, sensors, and observations” [32].

The first step in building the ontology is defining its architecture to specify the components and their relationships “within a system coupled with topology” [3]. Building the ontology is based on an incremental process starting by the identification of main concepts from the identified product–service development process. This process is followed by the extraction of all concepts, properties, and relationships from the common meta-model to form the backbone of the ontology. The last step is expanding the extracted concepts to a set of detailed taxonomies. The whole ontology is developed as a web ontology language (OWL) file with the Protégé tool.

To understand whether the ontology is coherent with the domain knowledge, industrial partners from the project check its formal structure, such as taxonomies and relationships. This validation has been done to finalize the sensor ontology for integration into the global ontology. In order to use the ontology from the knowledge repository, query algorithms are proposed to provide engineers with useful information. To investigate whether the ontology is able to fulfill information retrieval and interoperability needs, it has been tested in one specific scenario from the project.

### 3.2. The Global Model of the Proposed Ontology

**Figure 3:**
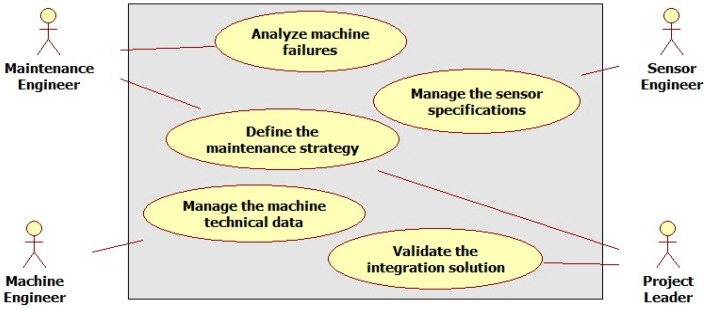
Use case diagram for maintenance service design.

To facilitate collaboration between several actors during maintenance service design and implementation, the proposed ontology is designed based on modular domain ontologies. To define the general structure of this ontology, the process of solution integration in the industrial context is reviewed. This process is highly dependent on a good matching of product components and sensors’ features specifications based on the objectives of the target maintenance service. A structured classification of these features is then required. The main involved actors and their roles during this process are (Figure 3):

(1) The maintenance engineer fixes the maintenance objectives and processes. He collaborates with the machine engineer during all analysis activities requested at the earlier stages of the sensor implementation process.
(2) The sensor engineer creates and manages sensor data and the technological specifications of the existing sensors in the repository. He contributes to the identification of the best sensor kits.
(3) The machine engineer uses legacy CAD (computer-aided design) tools to update and manage the necessary technical data of machine components in connection with the sensors. He provides his expertise on the nominal machine behavior as a contribution to the task of potential failures analysis.
(4) The project leader has the main role of supervising the interactions between involved engineers through the collaborative platform. He validates the maintenance strategy and coordinates the integration of the final solution of the target maintenance service.

**Figure 4:**
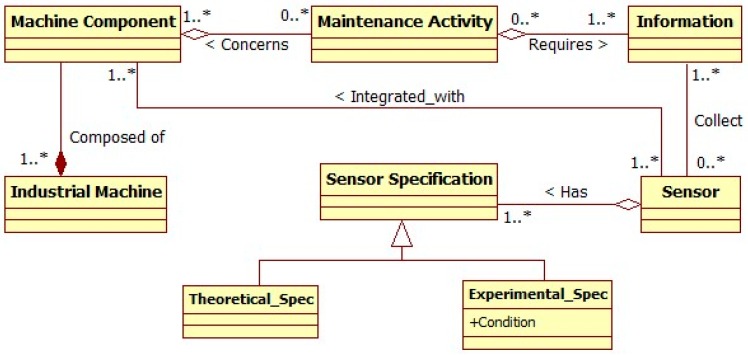
Meta-model heading the sensor ontology.

To build the generic sensor ontology, the global meta-model is extended and all concepts which have a direct effect on the sensor are extracted, connected, and classified. This allows for the identification of all ontology classes. Then, the relationships between classes are identified to form the final ontology structure. At the meta-level, as shown in Figure 4, the main classes heading the ontology structure are: Industrial machine, maintenance activity, information, sensor, and sensor specification. The sensor specification can be theoretical as defined by the sensor provider or experimental coming from real tests in specific conditions. For the genericity issue, the concept of maintenance activity is substituted to the concept of maintenance and maintenance service to focus on the operational level.

### 3.3. Sensor Ontology Structure

Based on the analysis of the main functionalities of maintenance, the proposed methodology is applied to support the information management of both the manufacturing process and maintenance from mechanical and electrical perspectives. The proposed modular ontology consists of semantic models of machines, sensors, and enabled services by these sensors.

**Figure 5:**
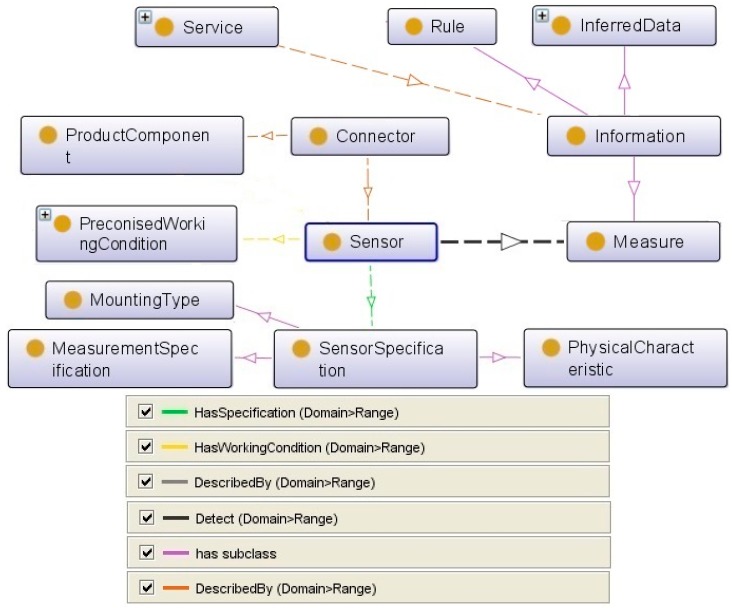
Sensing system ontology designed in protégé software.

The above defined main structure is modeled in protégé software (Figure 5) based on a sensor system according to the Semantic Sensor Network (W3C Incubator Group Report). Protégé software is a free, open source ontology editor and a knowledge management system. It provides a graphic user interface to define ontologies and includes deductive classifiers to validate the consistency of developed models and to infer new information based on the analysis of an ontology. Protégé manages ontology models in both Web Ontology Language (OWL) and The Resource Description Framework (RDF) files [33,34].

The class sensor takes a central place in the final ontology. It is one of the most critical tasks during the service design process to identify the best sensors for the related integration solution. To do so, a definition of the sensor is provided by a set of complementary taxonomies giving different points of view on the same sensor. The general logic behind this model is a scenario in which the requested service is analyzed then the information which is needed to fulfill the required maintenance activity is defined. This information describes the changes in the machine and its environment that the sensors detect. The sensor is described by a technological point of view and its connection with the machine’s components. The other important parameter is the specifications of the sensor, measurement, and working conditions. This class is detailed to define different sensors in detail. At the end, the ontology provides a complete list of well-described sensors with their detailed specifications and working conditions. This class’s attributes will be connected with the product, service, and information classes’ attributes to define the algorithm in Protegé.

As mentioned in Section 2.1, to cover the domain knowledge, it is necessary to abstract and generalize concepts into separate ontologies. According to this modular ontology strategy, each of these classes in the sensor ontology is detailed in their own ontology.

Considering all above, we defined various main classes in the sensor ontology as:

- Sensor (Technological Solution);
- Maintenance Activity as the Service;
- Information and Measure;
- Product/Machine Component;
- Sensor Specification;
- Mounting Type;
- Physical Characteristic;
- Measurement Specification;
- Working Condition; and
- Connection Constraint.

**Figure 6:**
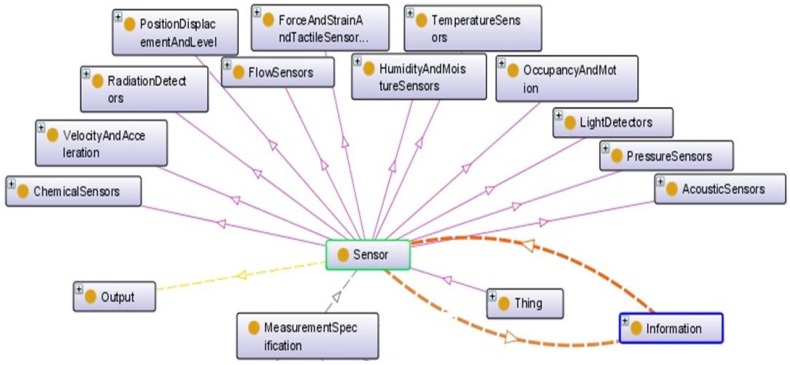
Sensor typology.

Using a sensor’s expert knowledge, the first step in the description of the sensor ontology is the classification of variety of sensor types. Each sensor is described by its input–output and its measurement specifications (Figure 6). This taxonomy is extracted from commercial portals and guidelines from sensor handbooks. This taxonomy is useful to extract the technical pre-defined specifications that suppliers or manufacturers provide in the sensors’ datasheets.

The sensor taxonomy is based on the related technological solution used to provide the desired measurement from the sensor of interest. It is a principle of the solution to provide the requested measurement by the sensor. This will help, for example, in the identification of the main technological constraints to be respected when selecting a sensor. It is also required by the compatibility verifier component to check if the used technologies allow the use of two different sensors (or more) in the same physical area of the product. The other sample is that each sensor can detect at least one change in the humidity or temperature of its environment. Some sensors can detect more than one kind of measure, such as humidity and temperature.

**Figure 7:**
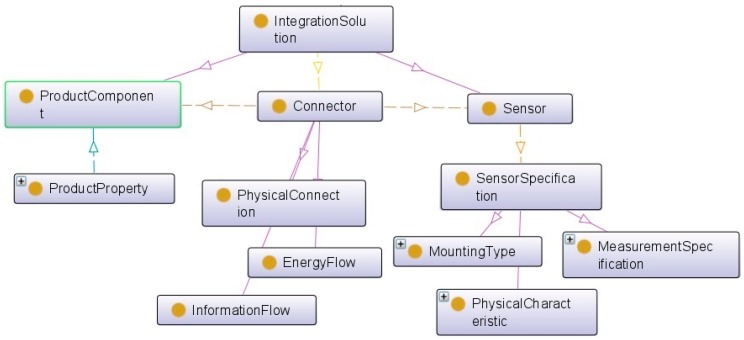
Ontology-based integration of sensor and machine components.

The process of providing the optimal integration solution starts with matching the sensors’ specifications and machine components’ specifications (Figure 7). This requires the implementation of specific connectors, and it is highly dependent on the machine’s properties. To support the connection process, the sensor ontology could also include the classification of connector types, such as physical connection, information flow, and energy flow.

The sensor specification gives the main characteristics of the sensor as described by the sensor provider. This will help, for example, in the rapid identification of possible connection constraints according to the mounting type. In addition, the concept of a sensor is described by the stimulus and additional properties. According to the W3C Semantic Sensor Network Incubator Group, Measure or Stimulus is “an event in the real world that triggers the sensor” [35]. We considered this measure as information detected by the sensor from its environment.

**Figure 8:**
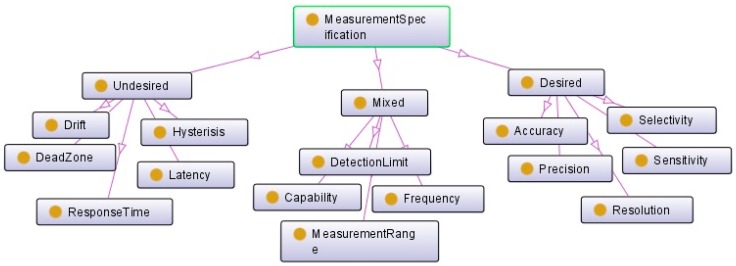
Measurement specification as key performance indicators of sensor.

A sensor may have some measurement capabilities describing the “capability of the sensor in various conditions” [35]. The measurement specification class can provide an indication about these capabilities. It describes the sensor’s operation range, which can be used in KPI (key performance indicator) as well as sensor selection. This will help the connection of sensor to the related categories of information for the rapid identification of suitable sensors for one maintenance activity. Three types can be distinguished based on the connection of their key indicators to the final performance of the sensor, namely: desired, undesired, and mixed (Figure 8). The first category includes the key indicators of the sensor’s measurement performance. The second one presents all factors that can reduce the performance, for example, the presence of dead zone or a high response time. The mixed indicators are the capacity, frequency, detection limit, and measurement range of the sensors. These indicators provide necessary information to choose the best sensor for different situations. For the same usage, the desired and undesired specifications indicate the weaknesses and advantages of each sensor.

**Figure 9:**
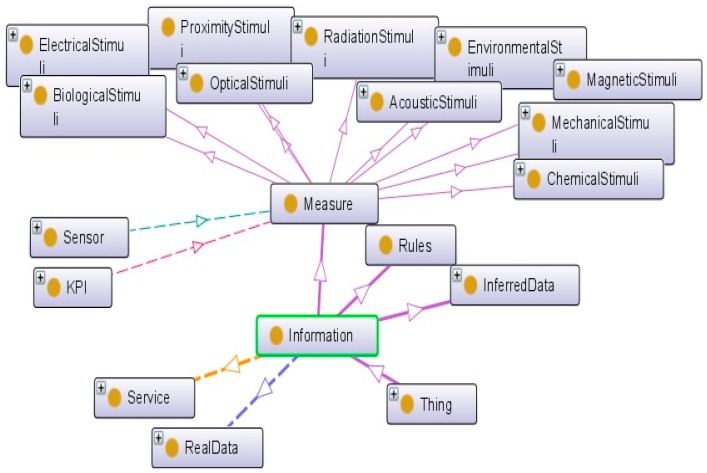
Information ontology.

For more details, the ontology is extended by including taxonomies of main concepts that have direct connection to the class sensor. For instance, each maintenance activity is defined by various information, namely: (1) measurement data collected from sensors; (2) rules supporting the interpretation of collected data regarding the characteristics of the business domain of interest (e.g., safe or hazard range); and (3) inferred data representing the results of the application on one rule Also, “Measurand” and “Stimuli” are defined as “detectable changes in the environment” [32] that “trigger the sensor” [33]. From the technological point of view, each sensor measures a special event. The classification of measurement types supports the rapid identification of the main attributes according to the measurement domain. This will help engineers to correctly introduce new sensors in the knowledge repository and as a result, identify more easily the best sensors at the conception stage (Figure 9).

**Figure 10:**
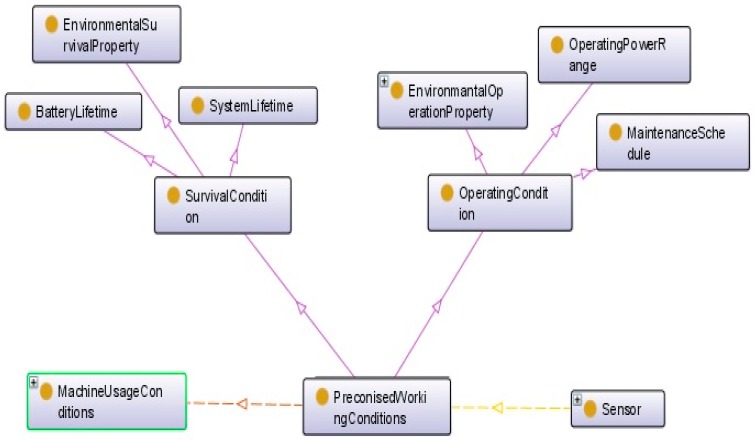
Sensor working condition specification.

The last category of modules describes the working condition that defines the environment where a sensor is embedded (Figure 10). Connecting sensor specifications and working conditions is one of the most important criteria to select the best sensor and the optimum solution. This is based on the matching between the nominal working conditions and the real working conditions of the target product. For example, matching the temperature or vibration which is produced by the machine with the temperature or vibration which a sensor can tolerate is an essential issue.

Finally, to integrate a service with a product, the connector plays the role of connecting the sensors, the equipment, and the product components. Connecting to the mounting type, this will help for example the identification of all constraints to be considered when fixing a sensor to a product component.

This section provided the detailed description of the ontology. The next section defines the industrial case in which the sensor ontology has been tested.

## 4. Application in Case Study

For validation perspective, the proposed ontology has been used to support the implementation of sensors for the condition-based maintenance of machine tools in High Speed Machining (HSM). Since the main objective is the validation of the proposal in terms of completeness and utility before its implementation in an industrial application, the manipulation of the ontology by engineers is ensured by Protégé software.

The case study focuses on spindles, which are the most efficient machine tool components (for productivity), but also the most damageable [36]. A spindle’s failure requires its replacement that stops the production for at least a few days, and the repair process is expensive. Consequently, the lifetime of a spindle that is too short has a noticeable impact on the cost of machined work pieces. These results have been issued from the research project UsinAE that has gathered end-users (in aeronautic sector) and manufacturers of machine tools and of spindles. The continuous monitoring of a spindle’s health is primordial to guarantee correct working conditions along the whole operational lifespan; to anticipate and plan maintenance activities; and to avoid an unproductive waste of time during curative maintenance actions.

**Figure 11:**
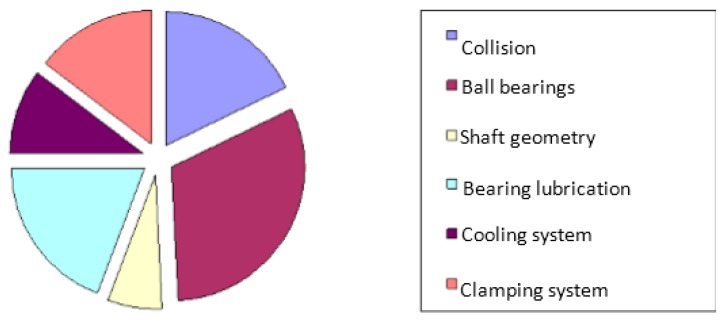
Failure ratios resulting from FMECA on damaged high speed machining (HSM) spindles.

In accordance with the sensor implementation process proposed in Figure 1, the study began with an FMECA. Around 250 diagnostic reports at spindle disassembly were analyzed and expert meetings were conducted. Every spindle failure mode was analyzed. Severity, occurrence, and potential detection of the failure or its cause led to a priority risk number. The main critical spindle component was revealed: it is the high precision ball bearings (Figure 11).

**Figure 12:**
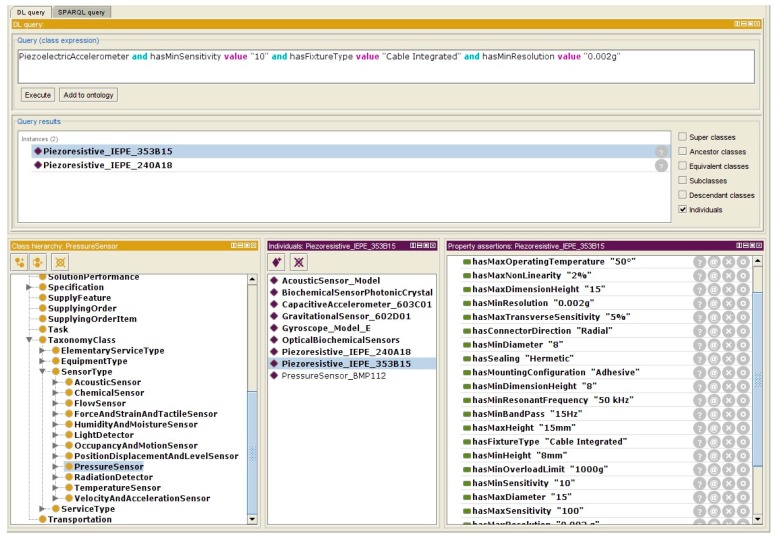
Protégé graphical interface for query management in the ontology.

One of the best technics for the condition-based maintenance of ball bearings is based on vibration monitoring. It is widely used for the maintenance of rotating machines. Following the proposed ontology, a short list of accelerometers was chosen from the repository in relation to their dimensions, sensitivity, band-pass, and other specifications. Firstly, based on his expertise, the designer of the maintenance service has defined the constraints and the required specifications for the sensor, which characterize the real context of use (Table 1). The ranking of a limited number of accelerometers was obtained based on the mapping of predefined requirements and sensors specifications defined by the sensors’ manufacturer (that had been implemented in the repository, Figure 12).

This result is obtained from a number of implemented instances of sensors and by queries on capabilities offered by the Protégé software. It eases the visualization of sensors’ characteristics during design activities. The ranking and analysis of queries results are fulfilled manually by the engineers based on their expertise. For testing issues, a list of the most-known sensor models in the studied domain has been created in the Protégé software as individuals (instances) of sensor sub-classes. Sensor research is fulfilled using “DL Query” facilities offered by Protégé as shown in Figure 12. The creation of new a attribute is fulfilled at the class level and spread to all existing instances.

As shown in Figure 12, the query is created according to the Table 1, which has been given by the machine’s engineers. As an example, one simple query is defined as “PiezoelectricAccelerometer and hasSensitivity value “10” and hasConnectorType value “Cable Integrated” and hasResonantFrequency value “70 kHz””. The Protegé retrieves two related sensors and shows the complete specifications. The engineer can choose the best sensor from the retrieved list.

**Figure 13:**
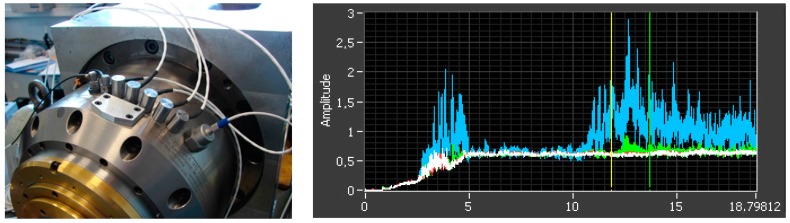
Test of accelerometers on a spindle test bed (**left**) and inappropriate results for most sensors (**right**: vibration level Vrms (mm/s) versus time (s)).

However, the measurements revealed that most of the commercial accelerometers are unsuitable for high speed spindles. Additional experimental work has been necessary to understand why and find a suitable sensor that fixes the issue. The study was conducted on a spindle test bed with a variety of accelerometers (Figure 13). From the acceleration measurement, the vibration level is usually assessed through the root mean square of vibration velocity, denoted Vrms, which is plotted on the graph. The 5 first seconds are a ramp-up until 24,000 revolutions per minute (RPM), followed by idle rotation. The colors refer to the signals of four different accelerometers. The blue one and the green one (to a lesser extent) are affected, whereas the white and red ones are more robust and measure steady values. It was found that the problem was due to piezoelectric saturation at high frequency. Based on the experimentation results, the selection of the best sensor is refined from the initial list proposed by the ontology-based repository.

In order to optimize the spindle monitoring quality, the sensors were integrated into the spindle, as close as possible to the bearings, both at the front and rear bearings, for higher sensitivity. The fixture was chosen by epoxy gluing, due the large bandpass required for a high speed spindle. Shielded cables were retained (in order to avoid electromagnetic interference) to conduct the signals from the spindle to the electrical cabinet after installation in the machine tool. NI9234 cards are used for the acquisition.

**Figure 14:**
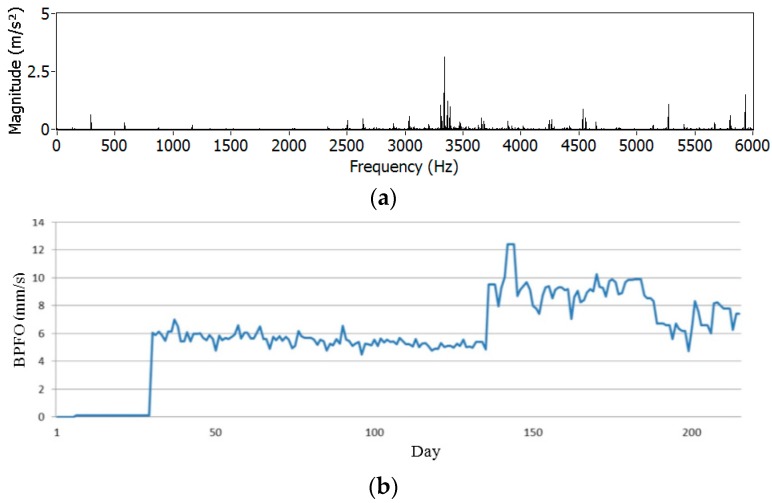
Example of frequency spectrum from a daily vibration signature of spindle (**a**) and evolution of ball pass frequency outer race (BPFO) monitoring criterion during 220 days (**b**).

Then, automatic measures of spindle vibration were daily programmed on the machine tool, in order to monitor the spindle’s condition (during idle rotations). Figure 14 presents an example of a vibration spectrum, where contributions due to bearing fault can be observed. The component at 3400 Hz is related to Ball Pass Frequency Outer race (BPFO), which reveals defects on the outer ring of the bearing.

At the end of the described process, the sensor-based maintenance solution and related experimental results were recorded in the sensor ontology-based repository, as a real sensor specification, providing an estimation of the sensor’s performance in specific working conditions. The structure of the proposed ontology is used as a guideline for the classification of captured data, making it easier to find the best solution in future similar cases. For this prototyping stage, this task is directly achieved in the Protégé tool. The ontology has been updated in Protegé for future use.

## 5. Conclusions and Discussion

The paper proposes a tailored ontology for the condition-based maintenance of industrial machines. The basic foundation of the ontology is extracted from the well-defined and developed ontology in the sensor engineering domain. Considering the special characteristics of condition-based maintenance, a dedicated sensor ontology has been developed for the maintenance of industrial machines. A new method to choose and implement the sensor, based on FMECA, is also presented.

While the taxonomies are extracted from sensor engineering, it is necessary to consider the machine-service structure and process in order to define the relationships. In this context, the first challenge is the customization process in the smart maintenance of machines. Indeed, there is a wide variety of sensors with different specific characteristics. Moreover, the performance of a given sensor can be largely affected by the application context and the working conditions. The second challenge is the choice of the suitable and robust sensor for the new condition-based maintenance of industrial machines.

The proposed ontology supports the classification of a wide panel of sensor data and their connection to real and pragmatic contexts of use. Protegé software provides formal mechanisms to support this task. The first experimentation of the proposal received interest from the industrial experts, who emphasized the permanent needs of different data, generally spread between different documents, software, and databases. Connecting data from the sensor, machine, and service to a common repository is of big interest.

Indeed, the resulting maintenance service finds satisfaction from maintenance experts who validate the technical choices taken during the design process and consequently demonstrate the efficiency of the proposed ontology. In addition, two questions are asked to the engineers in the feedback gathering process: do they find the information they need (e.g., sensor size, capacity)? Addtionally, do they feel that they save time when using such a tool? For the first question, the answer is positive. For the second, even if the engineers find interest in the solution, they agree that they lose considerable time to become familiar with the tool and to create the queries. This limitation is normal for this prototype stage because the use of a protégé solution is dedicated to experts on ontologies. Additional developments and experimentations are required to evolve the current repository from a prototype to an industrial decision-making application for CAD systems, taking into consideration ergonomic and intuitive aspects of the graphical interfaces.

The second limitation of the proposal at this step concerns the lack of matching with all business domains. The future work is the population of the proposed repository with additional sensor types to cover the wide variety of commercial sensors. Machine characteristics integration appears also as an interesting step to enhance the searching capacity of the proposed ontology.

## Figures and Tables

**Table 1:** Predefined requirements and specifications of sensor.

Specification		Attribute
Predefined Specifications (Ontology Classes)	Technology	Piezoelectric
	Class	IEPE
	Sensitivity	10 or 100 mV/g
	Measurement range	50 or 500 g
	Band-pass	15 Hz to 10 kHz
	Resonant frequency	>50 kHz
	Resolution	≤0.002 g
	Non-linearity	≤2%
	Transverse sensitivity	≤5%
	Overload limit	>1000 g
	Operating temperature	>50°
	Height	8–15 mm
	Diameter	8–15 mm
	Mounting configuration	Adhesive
	Type of electrical connector	Integrated cable
	Connector direction	Radial
	Sealing	Hermetic
**New Attribute**	Saturation issue	None
